# 

**Published:** 1865-12

**Authors:** 


### INDEX TO VOLUME VI.

Page.

Atropia and Morphia, Antagonism of, ... 689
Abnormal Fœtus, ... 390
Acute Gastritis, ... 139
Acute Orchitis, Treatment of, ... 47
American Medical Association, ... 57, 58, 113, 122, 172, 180, 252, 308, 315, 379, 391, 441, 586, 588
Arsenic Eating in Styria, ... 58
Asthma, Treatment of, ... 61
Alkaline Sulphites, On the Transformation of, ... 108
Army Medical Intelligence, ... 123
American Pharmaceutical Association, ... 171
American Dental Association, ... 573
Alphabetical Index to Braithwaite's Retrospect, ... 172
Apoplexy of the Spinal Gray Substance, ... 221
Assassination of President Lincoln, 310
Address by Dr. Bigelow, ... 392
" " Dr. N. S. Davis, ... 396, 430
Anæmia and Anæmiac Palpitation 735
Anstie, Francis E., On Stimulants and Narcotics, ... 743
Anæsthesia and Dr. W. T. G. Morton, ... 743
Army Surgeons Returned, ... 752
Atropia, Antagonistic Effects of, ... 756
Aneurismal Sac, Gangrene from Injection into, ... 758
Appliances for Invalids, ... 759
Air, Injection of, into Foul Abscesses, ... 760
Address of Dr. Allport before the American Dental Association, ... 573
Abortion, Will Oleum Tanaceti Cause, ... 446
Anæsthesia, Electrical, ... 444, 589

Bacteridia and Malignant Pustule, 368

Page.

Braithwaite's Retrospect of Practical Medicine and Surgery, ... 55
" " Index, ... 172
Bone, Regeneration of, ... 62
Belladonna in the Treatment of Iritis, ... 165
Black Troops and Consumption, ... 186
Beebread as a Diuretic, ... 557
Broncho-Pneumonia, Complicating Typhoid Fever, ... 712
Biddle, J. B., on Materia Medica, ... 742
Blood, Physiology of, ... 735

Catalytic Diseases, Therapeutic Value of Sulphites, ... 38
Chicago Medical College, ... 56, 117
Climate and Diseases of New Mexico, 65
Correspondence from New York, ... 79
Chicago Medical Society, ... 118, 172
Cerebro-Spinal Meningitis, ... 143, 208, 615
Citric Acid in Diabetes, ... 254
Cancer, Lecture on the Surgical Treatment of, ... 293
Chicago Academy of Sciences, ... 380
Compliment to an Army Surgeon, 381
Cases in Practice, ... 545
Cadaver, Antiseptic Injection for, 588
Continued Fever. By N. S. Davis, M.D., &c., ... 600
Cholera at Constantinople, ... 645
Cutaneous Medicine and Diseases of the Skin, ... 693
Chicago, Sanitary Condition of, ... 705
Clinical Cases in Medical Wards of the Mercy Hospital, ... 712
Chloroform, Use of Internally, ... 727
Classes, Longevity of, ... 753
Childs, Timothy, Death of, ... 753
Calabar Bean, Antagonistic to Atropia, ... 756
Choryza, Treatment of, ... 761

Page.
Davis, N. S., Report on Sanitary Measures in Chicago, 705
Davis, N. S., Clinical Lectures, 600, 694, 712
Davis, N. S., On Claims of Dr. W. T. G. Morton, 743
Dental Association, American, 573
Diphtheria, 1, 681
Diphtheria, Circular by Dr. Norton, 758
Deformities of the Feet, with Treatment, 125
Digitalis, its Physiological Effects, 188
Demand for Physicians, 190
DeWitt Co. Medical Society, 220, 566
Diuretics in the Treatment of Malarious Fevers, 269
Dysmenorrhœa, Slitting the Cervix in, 304
Dysentery, a New Remedy for, 609
Diary, Physician's Visiting List, &c., &c., 694
Duodeno-Hepatitis, or Acute Jaundice. By N. S. Davis, M.D., 694
Deaths—
Prof. D. L. McGugin, Keokuk, 570
Dr. J. Couper, New Castle, Del., 571
Dr. Melvin N. Rust, Surgeon of U. S. Vols., 572
Dr. E. A. Steele, 752
Dr. Timothy Childs, 753
Dr. William Hooker, 753

Excessive Use of Sugar, Effects of, 48
Epidemic Cerebro-Spinal Meningitis, 90
Essence of Turpentine in the headaches of Nervous Women, 187
Enlargement of the Prostate, 205
Epidemic. Plague in St. Petersburg, 249
Epidemics in Russia and North Germany, 301
Entozoa in Veal and Beef, 305
Eye, Diseases of, 321
Epidemic Erysipelatous Fever. By R. E. McVey, Waverly, Ill., 331
Explanation, 532, 635
Entozoa, in the Calf, 757
Electrical Phenomena in the Human Body, 759
Electrical Anæsthesia, 444, 589

Fibrous Tumors of the Uterus, On the Treatment of, 229
Fibrous Tumors, Treatment of, 236
Face, Partial Reconstruction of, 243

Page.
Fœtus, Abnormal, 390
Fox River Valley Medical Association, 533, 658
Food and the Teeth. By J. Paul, M.D., Trenton, N.J., 623, 661
Fever, An Eastern Antidote against, 599
Fever, Lectures on, 694
Fever, Typhoid, Clinical Remarks on. By N. S. Davis, M.D., 712
Fever, On the Treatment of, 54
Fever, Marsh, Pathological Appearances in, 718
Fossil Man, 761
France, Military Surgery in, 755

Gun-Shot Wounds and other Injuries of Nerves, 115
Glaucoma, Its Symptoms, Diagnosis, and Treatment, 116
Gastritis, Acute, 139
Gas, Nitrous Oxide, Properties of. By Geo. J. Ziegler, M.D., 742
Gangrene of the Hand, from Injections into an Aneurismal Sac, 785

Hospital Appointment, 121
Hospital Gangrene. By John E. Link, 147
Hospital, Pennsylvania, 253
Hand-Book of Skin Diseases, 306
Hospitals, Parisian, 381
Homœpathy, Surgeon-Gen. Barnes on, 442
Health of the Pacific Coast, 445
Hydrocele, Treatment of, 445
Hooker, Sir William, death of, 753
Human Body, Effects of Lightning on, 759

Illinois State Medical Society, 57, 121, 179, 272, 308, 585
Indian Ink, Substitutes for, 188
Illinois State Hospital for the Insane, Ninth Biennual Report, 243
Irritable Ulcers, Sour Milk to, 246
Inflammation, Lectures on, 694
Injections into Aneurismal Sac, 758
Invalids, Appliances for, 759
Injection, Danger of Subcutaneous, 760
Injection of Air into Abscesses and Fistulæ, 760

Letter from Dr. W. N. Cote, 44
London Lancet, 56, 308, 728, 763
Larynx of the Negro, 183
Letter from Liverpool, 214

Page.
Livingston, Dr., Contemplated visit to Africa, 317
List of Officers and Committees of the Amer. Med. Association for 1866, 420
Lithotrity, Proofs of its Success, 433
Letter from Dr. N. S. Davis, 437
Letter from Paris, 535, 653
Laryngoscope in Diseases of the Throat, 694
Longevity, in England and Wales, 762
Longevity of Classes, 753
Literature, Medical, in the South, 755

Military Surgery and Hygiene, A Treatise on, 54
Medical Lexicon, 115
Mortality of Chicago, 56, 119, 189, 316 443, 586, 636, 637, 754
Medical Intelligence, Foreign, 189
Medical Instruction, Summer, 247
Medical Students, French, 317
Medical Advertisements, Immoral, 532
Manual Army, Surgeons, 568
Medical Schools in Chicago, 569
Medical Colleges in Cincinnati, 635
Medical Colleges, 634
Medical Societies in Canada, 688
Medicine and Surgery, Practice of. By W. H. Byford, M.D., 692
Marsh Fever, Pathological Appearances Presented in, 718
Medical School at Cairo, Ill., 726
Meigs, J. Forsyth, on Pathological Appearances in Marsh Fever, 718
Merrill, A. P., on Chloroform Internally, 727
Morton, W. T. G., and Anæsthesia, 743
Morphia, Atropia and, Antagonism of, 689
Military Surgery in France, 755
Medical Department, Records of, 761
Malgaigne, Prof., 762
Mortality for October, 754
Medical Schools in the South, 753

Notice, 116
New Hospital for Women and Children, 120
New York Medical Journal, 173
New Remedies, A Report on, 285
New York State Lunatic Asylum, 307
Necrosis as an effect of Erysipelas, 338
Nose, Diseases of, 362
New Members Elected, American Medical Association, 417
New Dispensatory, 442

Page.
Onychia, Treatment of, 60
Obituary, 62, 247
Organs in the Human Subject unprovided with Nerves, &c., 124
Œsophagism, 253
Ophthalmology, 381
Ophthalmoscope, A Simple, 445
Orthopedic Surgery, Report on. By David Prince, M.D., 449
Ophthalmia, Gonorrhœal and Purulent. By J. S. Hildreth, M.D., 673
Orchitis Treatment of Acute, 47
Oleum Tanaceti, Will, Cause Abortion, 446

Pneumonia, Double, with Chronic Bronchitis, 36
Pyæmia, Two Cases of, 105
Permanganate of Potash as a Remedy, 111
Personal, 121, 381
Petroleum, Effects of, on Nervous System, 123
Purification of the Press, 173
Practical Items, 181
Pharmaceutists' and Druggists' Practical Receipt Book, 243
Public Address. By N. S. Davis, M.D., 257
Procidentia, A Radical Operation for, 307
Patents for Surgical Instruments, 340, 608
Peritonitis, Tubercular, 356
Peritonitis, Puerperal, 385
Papers and Reports from Special Committees Am. Med. Associa'n, 413
Puerperal Fever, complicated with Secondary Uterine Hemorrhage. By Swayne Wickersham, M.D., 549
Prescription Book, Physicians', 569
Premium Offered, 589
Poisoning, Case of, with Arsenious Acid. By R. C. Hamill, M.D., 643
Protoxide of Nitrogen, its Medical Properties and Applications, 694
Practical Pharmacy, 701
Practice, Cases in, 545
Physiology of Blood, 735
Pathological Appearances Presented in Marsh Fever, 718
Prize on Vaccino-Syphilitic Question, 762

Quinine, An Ounce at a Dose, 317
Quackery in Turkey, 432

Page.
Respiration after Decapitation ... 61
Rubeola complicated with Pneumonia. By J. P. Wardner, M.D. 77
Renal Calculi, ... 123
Remittent Ophthalmia—Strumous Ophthalmia, ... 151
Review of a Paper on the Unknown Functions of the Pancreas, ... 156
Recent Progress in Surgery, ... 172
Resolutions on the Death of Drs. Knight, Hooker, and Silliman, 421
Report of the Committee on the Case of M. A. Pallen, ... 427
Renewal of Life, ... 567
Remarks of Dr. N. S. Davis, to the Members of the American Dental Association, ... 576
Report on the Means of Improving the Sanitary Condition of Chicago, ... 705

Science of Light, Recent Advances in, ... 25
Scarlatina, Condition of the Stomach and Intestines in, ... 50
Syphilis, Recent Investigations upon the Subject of, ... 133
Sulphuric Ether vs. Chloroform, ... 176
Small-Pox, Protection from, ... 193
Surgical Pathology, Lectures on, ... 243
Small-Pox in Lahore, ... 303
Scrivener's Palsy, Lectures on, ... 342
Starvation of Prisoners, ... 371
Sanitary Commissions and Fairs, ... 376
State Medical Society, Illinois, ... 378
Soldiers' Home, ... 380
Speech of Dr. N. S. Davis, at Long Island, ... 424
Speech of the Governor of Massachusetts before the Amer. Medical Association, ... 426
Small-Pox in Algiers, ... 432

Page
Surgeons Wanted for the Army at Louisville, Tenn., ... 445
Surgeons for the Army, ... 532
Sanitary Condition of Chicago, from April 1st to Aug. 1st, 1865, 559
Scabies, Diagnosis and Treatment of, ... 583
Surgical Cases. By E. Andrews, M.D., ... 596
Schirrus, Case of. By R. S. Lewis, M.D., ... 641
Stomach, Diseases of, ... 693
Stricture of Urethra, Treatment of, 593

Typhus and Typhoid Fevers, Alcoholic Stimulants in, ... 82
Tumors, Destruction of, by Electricity, ... 123
Treatment of Squint by Operation, 168
Typhoid Fevers, Synopsis and Treatment of, ... 555
Typhoid Fever complicated with Broncho-Pneumonia. By N. S. Davis, M.D., ... 712
Treatment of Fever, On the, ... 54

Uterine Tumors, Treatment of, ... 129
Uterus, Expulsion of a Fleshy Mass from the, ... 541

Vaccine Report, ... 247
Vest-Pocket Medical Lexicon, ... 307
Varicose Veins, Obliteration of. By E. Andrews, M.D., ... 327
Volunteer Papers, (A.M.A.) ... 413

Weights and Measures used in Pharmacy, ... 185
Wanted, Medical Practice, ... 587

Zymotic and Constitutional Diseases, &c., On the Prevention of, 84

### BLISS & SHARP,

Wholesale & Retail

### DRUGGISTS AND CHEMISTS,

**144 LAKE STREET, CHICAGO,**

Keep constantly on hand a large assortment of

**PURE DRUGS FINE CHEMICALS,**

THAYER'S FLUID EXTRACTS & SUGAR-COATED PILLS'

**Keith's Preparations,**

Agents for the sale of

**Tiemann's Celebrated Surgical Instruments,**

Trusses, Elastic Stockings, Bandages, &c.

**Particular Attention Paid to Physicians' Orders.**

THE

### CHICAGO MEDICAL EXAMINER.

**N. S. DAVIS, M.D., EDITOR.**

A MONTHLY JOURNAL

DEVOTED TO THE

EDUCATIONAL, SCIENTIFIC, AND PRACTICAL INTERESTS

OF THE

MEDICAL PROFESSION.

The EXAMINER will be issued during the first week of each month, commencing with January, 1860. Each number will contain 64 pages of reading matter, the greater part of which will be filled with such contents as will directly aid the practitioner in the daily practical duties of his profession.

To secure this object fully, we shall give, in each number, in addition to ordinary original articles, and selections on practical subjects, a faithful report of many of the more interesting cases presented at the Hospitals and College Cliniques. While aiming, however, to make the EXAMINER eminently practical, we shall not neglect either scientific, social, or educational interests, of the profession. It will not be the special organ of any one institution, society, or clique; but its columns will be open for well-written articles from any respectable member of the profession, on all topics legitimately within the domain of medical literature, science, and education.

Terms, $3.00 per annum, invariably in advance.

